# Factors associated with compassion fatigue among ICU nurses in Greece

**DOI:** 10.1186/cc9909

**Published:** 2011-03-11

**Authors:** P Mangoulia, G Fildissis, E Koukia, G Alevizopoulos, T Katostaras

**Affiliations:** 1 National and Kapodistrian University of Athens, Greece

## Introduction

ICU nurses work in a demanding environment and they are repetitively exposed to traumatic situations and stressful events. There is a growing interest in the phenomenon of compassion fatigue (CF) and its impact on healthcare professionals; however, its impact on ICU nurses is basically unknown. The primary aim of this study was to investigate the risk for CF (the trauma suffered by the helping professional) and burnout (BO - emotional exhaustion, depersonalization and reduced sense of personal accomplishment), and the potential for compassion satisfaction (CS - the fulfillment from helping others and positive collegial relationships) among ICU nurses. An additional goal was to test the relationship between nurses' characteristics (demographic and occupational) and CF risk.

## Methods

The Professional Quality of Life Scale (ProQOL R_IV, CF, BO and CS subscales) and a demographic tool were distributed to 335 ICU nurses in 22 public hospitals in the Athens greater area, Greece.

## Results

Findings revealed that 57.9% of ICU nurses are at the high level of risk for CF and 56.1% are at the high level of risk for BO, while 61.5% of participants reported low potential for CS. Female nurses (*P *= 0.016), with low income (*P *= 0.041), married (*P *= 0.001) or widowed (*P *= 0.023), who work as assistant nurses (*P *= 0.014) and also registered nurses with Master of Science (*P *= 0.008) or Nursing Specialty (*P *= 0.003) were found to have higher risk for CF. Additionally, higher risk for CF had also participants who characterized their relationship with their colleagues as neutral (*P *= 0.001) or bad (*P *= 0.030), believed that the staff work sometimes as a team (*P *= 0.016), spend 26 to 100% of their work time in direct contact with the patients (26 to 50%: *P *= 0.001, 51 to 75%: *P *= 0.043, 76 to 100%: *P *= 0.024) and described their mental health as poor (*P *= 0.001), average (*P *< 0.001) or good (*P *< 0.001). Nurses who want to retain in the ICU (*P *= 0.003) and those who want to leave the hospital in few years (*P *= 0.005) were associated with lower risk for CF.

## Conclusions

The high prevalence of CF in our sample indicated that large numbers of ICU nurses may be experiencing these negative effects. Knowledge of CF-related variables may help healthcare organizations identify nurses at risk, provide intervention strategies to maintain healthy outcomes for nurses and increase job satisfaction.
